# Going beyond ensemble average angular cross-correlation analysis

**DOI:** 10.1107/S205225252200656X

**Published:** 2022-06-29

**Authors:** Espen Drath Bøjesen

**Affiliations:** a Interdisciplinary Nanoscience Center and Centre for Integrated Materials Research, Aarhus University, Gustav Wieds Vej 14, DK-8000 Aarhus C, Denmark

**Keywords:** X-ray scattering, X-ray cross-correlation analysis, structure determination, crystalline defects

## Abstract

Commentary is given on a paper [Lapkin *et al.* (2022). *IUCrJ*, **9**, 425–438] reporting the application of angular X-ray cross-correlation analysis to the scattered intensity distribution measured in 3D reciprocal space from a single-crystalline sample.

Analysis of angular correlations of scattering intensities has, albeit initially only sporadically, been used as a tool for studying materials structure dating back to the 1970s (Kam, 1977 ▸; Clark *et al.*, 1983 ▸). However, since the seminal work by Wochner *et al.* (2009 ▸), angular cross-correlation analysis (ACCA) has experienced somewhat of a renaissance due to advances in instrumentation and computing power. The advent of third- and fourth-generation synchrotron sources, free-electron lasers, modern electron microscopes, novel detector technologies, and improved computing capabilities have led to a renewed interest in ACCA. Recent studies include expansion of the underlying theory (Altarelli *et al.*, 2010 ▸; Martin, 2017 ▸) and a plethora of examples where ACCA has been central to revealing novel insights into, for example, the mechanical stability of colloidal glasses (Liu *et al.*, 2022 ▸), the structural nature of metallic glasses (Liu *et al.*, 2013 ▸), defects in disordered carbons (Martin *et al.*, 2020 ▸), and even protein structures (Adams *et al.*, 2020 ▸).

In the current issue of **IUCrJ** , Lapkin *et al.* (2022 ▸) propose a novel application of ACCA – namely expanding its applicability to facilitate analysis of single-crystal X-ray scattering data collected from colloidal single crystals. This tactic breaks with more commonly adapted approaches to ACCA, which rely on collecting vast amounts of X-ray or electron scattering patterns from randomly oriented individual samples or from multiple different locations in one individual sample (possible with scanning X-ray or scanning electron scattering approaches). Both these tactics assume that by collecting enough individual scattering patterns the entire 3D reciprocal space volume will be sampled. This notion is reasonable for structurally homogeneous samples without any significant degree of preferred orientation, such as glassy materials, but may fail for more ordered samples.

The new scheme devised by Lapkin *et al.* avoids the need for complex iterative phase-retrieval algorithms while still providing critical qualitative insights to the real-space structure of a single-crystalline grain. By studying individual crystals, insights into potential structural variations across a sample (*i.e.* a collection of crystals from a ‘synthesis’) may be accessed. This contrasts previous ACCA of scattering data, which generally provides insights into ensemble average structure. The original methodology thus facilitates *quick-and-easy* assessment of structural features in colloidal crystals. In the paper, Lapkin *et al.* nicely demonstrate the feasibility of their approach by presenting analyses of both simulated as well as experimental X-ray scattering data to reveal various types of stacking disorder in colloidal crystals. The ability to easily judge the type and approximate concentration of such defects is where this method really excels.

The addition of this tool to the ever-expanding toolbox of scattering-based structural analysis is a timely reminder of the importance of considering the differences between studies of the ensemble average structure of a sample and studies of spatial variations of local structure in a sample. Within the field of X-ray scattering (not counting single-crystal studies) there has been a tendency to focus on the former to provide important structural insights with impressive statistical significance. This statistical potency, however, inadvertently comes with a lack in sensitivity towards often property defining manifestations of structural heterogeneity. Similarly, methods that only study very small sample volumes, *e.g.* electron microscopies, lack the statistical gravitas of ensemble average methods. As previously mentioned, vast improvements in experimental capabilities now finally enable detailed studies of structural heterogeneity at multiple lengths scales. Along these lines, there lies great potential in exploring the combination of the methodology from Lapkin *et al.* with already established ‘ensemble average’ based ACCA techniques to get *the best of both worlds.*

Finally, the proof-of-concept study presented by the authors opens attractive new avenues. For one, the newly established tactics can be used in their current state to reveal the structural origin of relevant materials problems – including *in situ* or *operando* studies. Furthermore, expanding the capabilities of the presented analysis approach to apply to structures of other length scales than colloidal crystals is an exciting direction for the community to explore. For example, it would be interesting to investigate the possibility of studying assemblies of nanosized (2–4 nm) atomic clusters into hierarchical structures or even the atomic structure of nanosized single crystals by adapting the method from Lapkin *et al.* to be suitable for analysis of electron scattering data.
